# Arabidopsis apoplastic fluid contains sRNA- and circular RNA–protein complexes that are located outside extracellular vesicles

**DOI:** 10.1093/plcell/koac043

**Published:** 2022-02-16

**Authors:** Hana Zand Karimi, Patricia Baldrich, Brian D Rutter, Lucía Borniego, Kamil K Zajt, Blake C Meyers, Roger W Innes

**Affiliations:** Department of Biology, Indiana University, Bloomington 47405, Indiana, USA; Donald Danforth Plant Science Center, St Louis 63132, Missouri, USA; Department of Biology, Indiana University, Bloomington 47405, Indiana, USA; Department of Biology, Indiana University, Bloomington 47405, Indiana, USA; Department of Biology, Indiana University, Bloomington 47405, Indiana, USA; Donald Danforth Plant Science Center, St Louis 63132, Missouri, USA; Division of Plant Sciences, University of Missouri-Columbia, Columbia 65211, Missouri, USA; Department of Biology, Indiana University, Bloomington 47405, Indiana, USA

## Abstract

Previously, we have shown that apoplastic wash fluid (AWF) purified from Arabidopsis leaves contains small RNAs (sRNAs). To investigate whether these sRNAs are encapsulated inside extracellular vesicles (EVs), we treated EVs isolated from Arabidopsis leaves with the protease trypsin and RNase A, which should degrade RNAs located outside EVs but not those located inside. These analyses revealed that apoplastic RNAs are mostly located outside and are associated with proteins. Further analyses of these extracellular RNAs (exRNAs) revealed that they include both sRNAs and long noncoding RNAs (lncRNAs), including circular RNAs (circRNAs). We also found that exRNAs are highly enriched in the posttranscriptional modification N^6^-methyladenine (m^6^A). Consistent with this, we identified a putative m^6^A-binding protein in AWF, GLYCINE-RICH RNA-BINDING PROTEIN 7 (GRP7), as well as the sRNA-binding protein ARGONAUTE2 (AGO2). These two proteins coimmunoprecipitated with lncRNAs, including circRNAs. Mutation of *GRP7* or *AGO2* caused changes in both the sRNA and lncRNA content of AWF, suggesting that these proteins contribute to the secretion and/or stabilization of exRNAs. We propose that exRNAs located outside of EVs mediate host-induced gene silencing, rather than RNA located inside EVs.

## Teaser

The apoplast of Arabidopsis leaves contains diverse small and long-noncoding RNAs, including circular RNAs, that are bound to protein complexes and are located outside extracellular vesicles.

IN A NUTSHELL **Background:** To prevent infection by disease-causing microbes, plants secrete diverse antimicrobial compounds into their extracellular spaces. Included among these compounds are small RNA molecules 21-24 nucleotides in length (sRNAs) that can be taken up by microbes. These sRNAs are thought to cause the destruction of messenger RNAs having complementary sequences. The processes by which sRNAs are secreted, how they are protected from degradation, and how they are taken up by pathogenic microbes are all poorly understood.

**Questions:** Although extracellular sRNAs have been shown to copurify with extracellular vesicles (EVs), their exact location had not been clearly established. Are sRNAs located inside or outside EVs? Also, do plants secrete RNAs longer than 24 nucleotides?

**Findings:** We isolated EVs from the extracellular spaces of Arabidopsis leaves and then treated these preparations with RNase A to degrade naked RNA or with protease plus RNase A to degrade RNA protected by proteins. Our analyses revealed that sRNAs are associated with protein complexes that are located outside EVs. Significantly, we found that Arabidopsis secretes both sRNAs and much longer RNAs, ranging from 30 to over 500 nucleotides in length. These longer RNAs do not code for proteins, and many have a circular structure. Notably, both these long noncoding RNAs and the sRNAs were found to be highly enriched in a posttranscriptional modification known as N6 -methyladenine. We speculate that this modification might be required for secretion of RNA.

**Next steps:** The discovery that plants secrete long noncoding RNAs, including circular RNAs, was unexpected and raises the question as to why. Do they play a role in cell-to-cell communication within the plant? Are they an important component of the immune system? How are these RNAs secreted and what are the roles of RNA-binding proteins and posttranscriptional modifications in this process?

## Introduction

The apoplast is the extracellular space outside the plasma membrane of plant cells that comprises the cell wall, the xylem, and any space between cells (Steudle, 1980; Guerra-Guimarães et al., 2016). Apoplastic fluid contains water, sugars, amino acids, cell wall modifying enzymes, growth regulators, and diverse stress-related proteins (Guerra-Guimarães et al., 2016; Huber and O'Day, 2017; Narula et al., 2020; Wang and Dean, 2020; Wang et al., 2020). Recently, we and others have shown that apoplastic fluid also contains extracellular vesicles (EVs) that carry defense-related proteins and small RNAs (sRNAs) (Rutter and Innes, 2017; Cai et al., 2018a; Baldrich et al., 2019; He et al., 2021). The role of EVs in plant–microbe interactions is thus an intriguing and active area of investigation.

It is known that sRNAs from both plants and pathogens can hijack microbe or host RNA interference pathways to induce trans-kingdom gene silencing (Weiberg et al., 2013; Niu et al., 2015; Wang et al., 2017; Hou et al., 2019; Huang et al., 2019; Schaefer et al., 2020). Expression of plant sRNAs that target pathogen genes has been used to confer resistance to diverse fungal, nematode, and insect species (Nowara et al., 2010; Koch et al., 2013; Mamta et al., 2016; Qi et al., 2019). However, it is not clear how the sRNAs are transferred between plant and pathogen cells. To avoid degradation, it is speculated that these extracellular RNAs (exRNAs) need to be either tightly associated with RNA-binding proteins or to be encapsulated within EVs (Rutter and Innes, 2017; Koch and Wassenegger, 2021). However, whether EVs and/or RNA-binding proteins are required for RNA secretion or movement within the apoplast is unclear and still under investigation.

Previously, we have reported that apoplastic wash fluid (AWF) of Arabidopsis (*Arabidopsis thaliana*) contains diverse species of sRNAs, including microRNAs (miRNAs), small interfering RNAs (siRNAs), and a previously overlooked class of tiny RNAs (tyRNAs; 10–17  nt) with unknown functions (Baldrich et al., 2019). In that study, we showed that apoplastic tyRNAs copurified with EVs when using a density gradient. Notably, siRNAs and miRNAs were largely missing from density gradient-purified EVs, although they were present in total AWF. These observations suggested that EVs may not be the primary carrier of apoplastic siRNAs and miRNAs (Baldrich et al., 2019). In support of this hypothesis, analysis of apoplastic siRNAs derived from transgenic expression of a hairpin RNA in Arabidopsis revealed that >70% of these were located outside EVs (Schlemmer et al., 2021).

Although density gradient centrifugation is a preferred method for obtaining highly pure EV preparations (Rutter and Innes, 2020), it is still possible for large RNA–protein complexes to copurify with EVs, or RNAs to adhere to the surface of EVs, thus most work published to date, including our own, has not established whether plant EV-associated RNAs are located inside or outside EVs. To eliminate extravesicular RNA–protein complexes and RNA attached to the surface of EVs, it is necessary to treat purified EVs first with proteases to remove any RNA-binding proteins and then with RNase to degrade the released RNAs (Rutter and Innes, 2020).

Recently, He et al. (2021) identified several RNA-binding proteins in the apoplast of Arabidopsis leaves that might be responsible for loading sRNAs into EVs, including ARGONAUTE1 (AGO1), ANNEXIN1 and 2 (ANN1 and ANN2), and RNA HELICASE11 and 37 (RH11 and RH37). Protease protection assays indicated that these proteins are all located inside EVs. However, this work did not include a protease plus RNase treatment, thus did not distinguish between sRNAs located outside EVs in RNA–protein complexes versus sRNAs located inside EVs (He et al., 2021). Similarly, Cai et al. (2018a, 2018b) used micrococcal nuclease treatment to show that sRNAs that had copurified with EVs from Arabidopsis leaves were protected from degradation. However, because the lack of prior protease treatment likely left RNA–protein complexes intact, this analysis also did not distinguish between sRNAs located in RNA–protein complexes versus those located inside EVs.

Although plant EVs have only been reported to contain sRNAs and tyRNAs, mammalian EVs have been reported to carry sRNAs as well as long noncoding RNAs (lncRNAs), including circular RNAs (circRNAs) (Xu et al., 2020b). circRNAs are covalently closed, single-stranded circles derived from back-splicing reactions of RNA polymerase II transcripts, whereby a splice donor site at the 3′-end of an exon fuses to a splice acceptor site at the 5′-end of the same exon, or another upstream exon (Fu and Ares, 2014; Wang et al., 2021). circRNAs have been shown to play a regulatory role in multiple biological processes, including immune responses in both mammalian and plant systems (Hu et al., 2019; Mahmoudi et al., 2019; Fan et al., 2020; Zhang et al., 2020b). One mechanism by which circRNAs are thought to regulate gene expression is through attracting both miRNAs and RNA-binding proteins, and thereby sequestering them. Such sequestration will impact RNA transcription, splicing, and translation (Hansen et al., 2013; Jeck and Sharpless, 2014; Bose and Ain, 2018; Panda, 2018). Fan et al. (2020) demonstrated that circRNAs from rice (*Oryza sativa*) are involved in immune responses to the fungal pathogen *Magnaporthe oryzae*. Several circRNAs in rice leaves were detected only upon infection with *M. oryzae*. Furthermore, this work showed that overexpression of one specific circRNA enhanced rice immunity to *M. oryzae* (Fan et al., 2020), indicating that circRNAs may represent an important component of plant immune systems. However, whether circRNAs are secreted by plant cells, as they are by mammalian cells, has not yet been reported.

To understand the possible function of exRNAs in plants, we analyzed the sRNA and circRNA content of Arabidopsis apoplastic fluid both inside and outside EVs, as well as the RNA-binding proteins associated with these RNAs. Our data reveal that apoplastic fluid contains diverse RNA species, including sRNAs and lncRNAs (100 >500 nt), many of which appear to be circRNAs. The great majority of both sRNAs and lncRNAs were found to be located outside EVs. However, this extravesicular RNA is protected against degradation by RNases via association with RNA-binding proteins. The presence of abundant extravesicular sRNA- and circRNA–protein complexes in the apoplast suggests that these RNAs may play a central role in plant–microbe interactions and also contribute to host-induced gene silencing.

## Results

### The majority of apoplastic sRNAs are located outside EVs

Our previous analyses of sRNAs associated with density gradient-purified EVs revealed that EVs contain relatively few RNAs in the 21, 22, and 24 nucleotide (nt) size range, and instead are highly enriched in shorter RNAs that are 10–17 nt in length, and have been termed tyRNAs (Baldrich et al., 2019). Those analyses, however, did not assess whether these tyRNAs were located inside or outside the EVs and did not include any apoplastic sRNAs that pelleted at 40,000 *g* but that did not copurify with EVs in the density gradient.

To assess whether apoplastic fluid contains RNA-associated particles other than EVs, we generated sRNA libraries from pellets obtained after centrifuging AWF at 40,000 *g* for 1 h (P40 pellets; see “Materials and methods”). The P40 pellets contained a mixture of particles, including EVs. To distinguish between RNA located inside EVs from RNA located outside EVs, we treated P40 pellets with trypsin plus RNase A, which should eliminate RNA associated with proteins located outside EVs, while leaving RNAs located inside EVs intact. As controls, we treated pellets with just the buffer or with RNase A alone. The latter should degrade free RNA but not RNA bound to proteins or located inside EVs.

**Figure 1:**
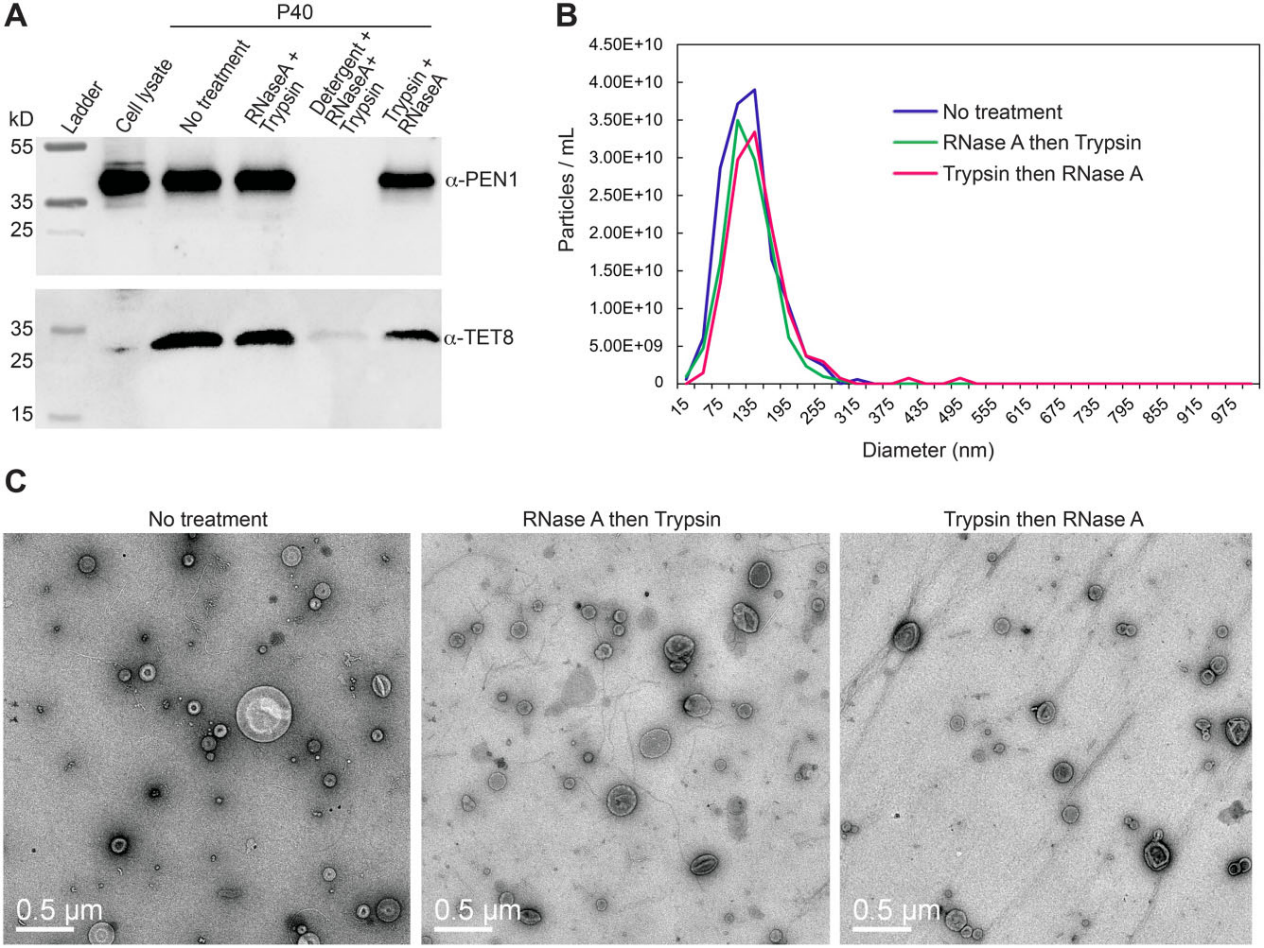
Neither trypsin nor RNase A treatment disrupts EVs. A, Protease protection assays of EV cargo proteins PEN1 and TET8. The P40 fractions were subjected to the indicated treatments and then analyzed by immunoblot analysis using anti-PEN1 and anti-TET8 antisera. RNase A and trypsin treatments were performed consecutively in the order indicated for each lane (see “Materials and methods” for details). PEN1 and TET8 were eliminated only when detergent (1% Triton X-100) was included in the mixture, indicating that EVs remained intact when treated with RNase A and trypsin in the absence of detergent. B, Nanoparticle tracking analysis of P40 fractions. Graphs show concentration and size distributions of particles in the three samples. The indicated treatments had no significant effect on these parameters. C, TEM images of negatively stained P40 fractions following the indicated treatments. The bean bag-like particles are EVs. Bars = 0.5 μm.

Importantly, these treatments do not disrupt EVs, as assessed by protease protection assays, nanoparticle tracking and negative stain transmission electron microscopy (Figure 1). For the protease protection assays, we assayed the EV proteins PENETRATION1 (PEN1) and TETRASPANIN8 (TET8), which are believed to mark different classes of EVs that could potentially carry different RNA cargos (He et al., 2021). Both markers were pelleted at 40,000 *g* and both were protected against digestion by trypsin, even after treating P40 pellets with RNase A first (Figure 1). We thus conclude that RNA carried in either EV type should be protected against RNase A digestion, whether or not the pellets are treated with trypsin first.

**Figure 2:**
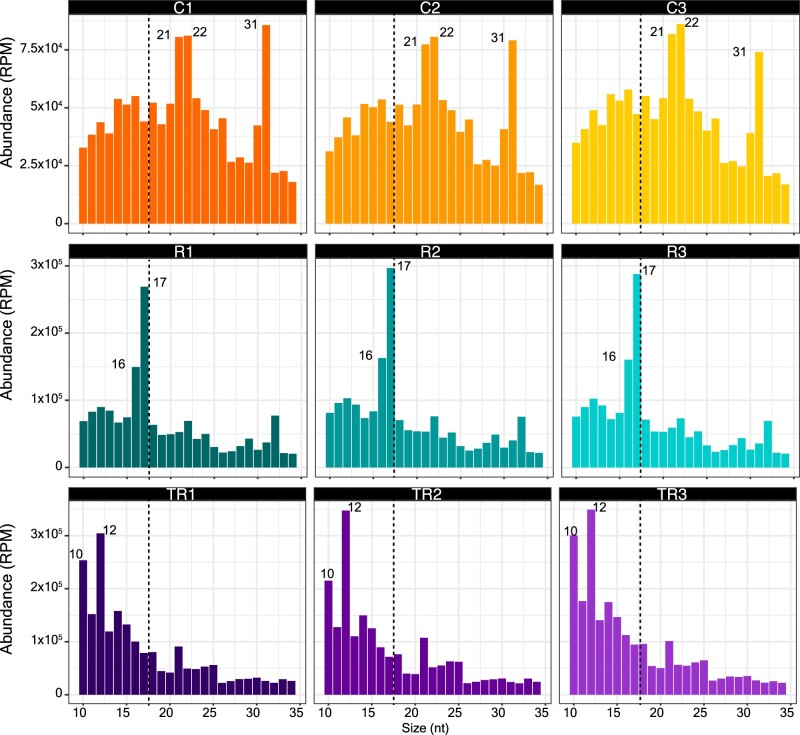
The majority of apoplastic sRNAs are located outside EVs. Size distribution of P40 sRNAs mapping to the Arabidopsis genome (TAIR version 10). The abundance of each size class was calculated for each P40 treatment: control (C1–C3), RNase A only (R1–R3), and trypsin plus RNase A (TR1–TR3). The *x*-axis indicates the sRNA size and the *y*-axis indicates its abundance in RPM mapped reads. Shown are data from three independent biological replicates, with each replicate derived from AWF pooled from 24 Arabidopsis plants. Note the depletion of 21-, 22-, and 31-nt RNAs following treatment either with RNAse A alone or with trypsin plus RNase A, indicating that these size classes were mostly found outside EVs.

Separate sRNA-seq libraries were generated from each of three biological replicates of each treatment (nine libraries in total) and sequenced using an Illumina NextSeq platform. We observed that the distribution of read lengths was consistent between replicates, but substantially different between treatments (Figure 2). Control samples displayed predominant peaks at 21, 22, and 31 nt, whereas samples treated with RNase A alone displayed peaks at 16 and 17 nt. Trypsin plus RNase A treated samples displayed peaks at 10 and 12 nt. These results are consistent with our previous analyses of density gradient-purified EVs in that EVs appear to contain very few 21-, 22-, or 24-nt sRNAs but are enriched in tyRNAs. Significantly, these results reveal that the apoplast contains large amounts of 21-, 22-, and 31-nt RNAs that are located outside EVs and bound to particles of some sort.

**Figure 3:**
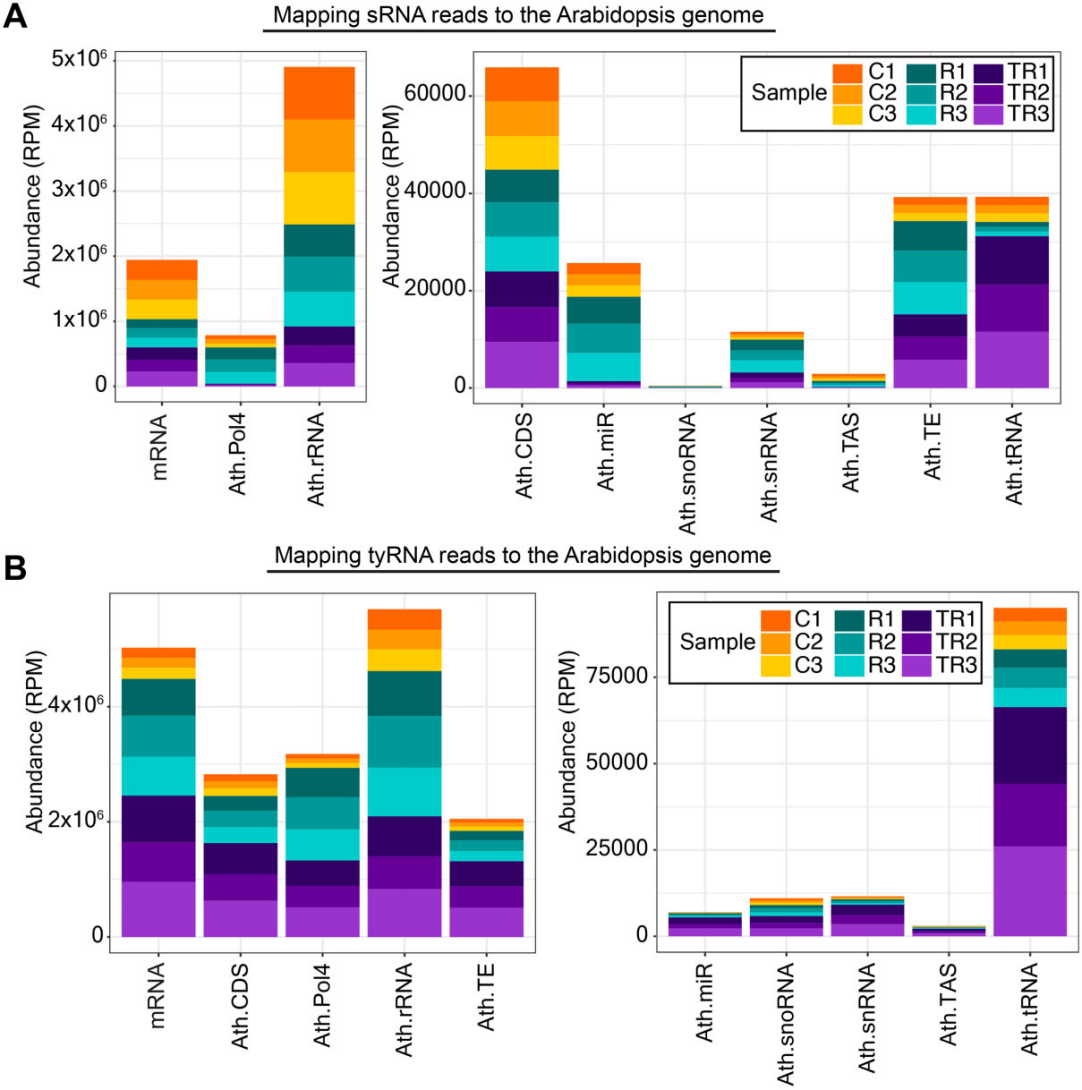
Apoplastic sRNAs are derived from diverse sources. A, Specific subclasses of sRNAs are protected by proteins. sRNAs that mapped to the genome were categorized by origin and plotted by relative abundance in RPM. Compared to controls (C1–C3), treatment with RNase A alone (R1–R3) increased the relative proportion of Pol IV-, miRNA-, snRNA- and TE-derived sRNAs, whereas treatment with trypsin plus RNase A (TR1–TR3) decreased their relative proportion. This indicated that the majority of these sRNAs are protected by protein and are located outside EVs. snoRNA, small nucleolar RNA; TAS, trans-acting siRNA. B, tyRNAs are mostly located inside EVs. tyRNAs that mapped to the genome were categorized by origin and plotted by relative abundance, as with the sRNAs above. All categories of tyRNAs increased in relative abundance upon treatment with trypsin plus RNase, indicating that they were protected against trypsin plus RNase treatment and hence are mostly located inside EVs. For both panels, the *x*-axis indicates the RNA source, and the *y*-axis indicates its abundance in RPM mapped reads. Data from three independent biological replicates (AWF pooled from 24 plants in each replicate) are stacked together in a single bar plot and color-coded as shown in the legend.

To further understand the nature of apoplastic sRNAs and tyRNAs, we analyzed their origin. We observed that most of the sRNA reads originated from rRNAs, mRNA, and products that were dependent on RNA polymerase IV (Pol IV) (Figure 3). We also observed that the treatment with RNase A and trypsin had a different impact on each RNA category. Although relative representation of mRNA and rRNA categories remained fairly constant after different treatments, the representation of Pol IV-, miRNA-, small nuclear RNA (snRNA)-, and transposable element (TE)-derived sRNAs increased after RNase treatment and decreased after trypsin plus RNase A treatment (Figure 3A). This pattern suggested that Pol IV-, miRNA, snRNA-, and TE-derived sRNAs were mostly located outside EVs but were protected from degradation due to association with proteins. In contrast, the relative amount of tRNA-derived sRNAs decreased after RNase treatment but increased after trypsin plus RNase treatment, suggesting that tRNA-derived sRNAs were present in the apoplast as unprotected RNAs outside EVs, as well as inside EVs. In the case of tyRNAs, we observed an increase in all categories after trypsin plus RNase treatment (Figure 3B). These patterns support our previous conclusion that tyRNAs are highly enriched inside EVs (Baldrich et al., 2019).

We further analyzed these sRNA-seq data by plotting the read-length distributions as a function of their origins (Supplemental Figure S1). As expected, read lengths for miRNAs and trans-acting siRNAs (tasiRNAs, TAS) displayed sharp peaks at 21 nt. Notably, this size distribution was not altered by treatment with RNase A alone, whereas treatment with trypsin plus RNase A eliminated the 21-nt peaks, leaving a peak at 10–12 nt. These observations further support our conclusion that sRNAs are primarily located outside EVs and are protected by RNA binding proteins, whereas tyRNAs are located inside EVs.

Supplemental Figure S1 also revealed that the peak at 31 nt observed in Figure 2 was almost entirely due to transcripts that overlap known Pol-IV-dependent 24-nt siRNAs (Zhou et al., 2018). Notably, this peak was eliminated by treatment with RNase A alone, leaving a peak at 16–17 nt. This observation suggests that these Pol IV-dependent transcripts are also located outside EVs but are only partially protected by RNA binding proteins. The observation that these transcripts are mostly 31 nt rather than 24 nt suggests that they are derived from precursor RNAs that did not complete maturation into 24 nt siRNAs by DICERLIKE 3 (DCL3) (Blevins et al., 2015).

### A small subset of miRNAs is enriched inside EVs

**Figure 4:**
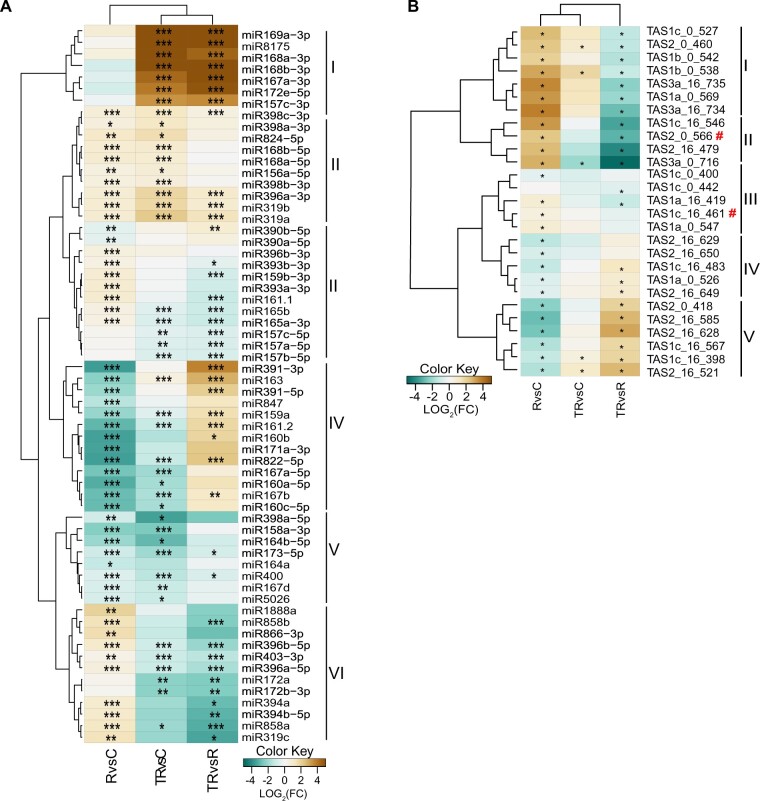
Apoplastic sRNAs are mostly found outside EVs. A, Apoplastic miRNAs having a minimum abundance of one RPM in at least one treatment and showing differential accumulation in at least one comparison were grouped into six clades based on their relative abundance following three different treatments: RNase A alone (R), trypsin plus RNase A (TR), or a negative control with no treatment (C). The heat map indicates enrichment (brown) or depletion (teal) in one treatment compared to another. B, Apoplastic tasiRNAs having a minimum abundance of five RPM in at least one treatment and showing differential accumulation in at least one comparison were grouped into five clades based on their relative abundance. Red hashtags indicate tasiRNAs previously reported to mediate silencing of genes in the fungus *Botrytis cinerea* (Cai et al., 2018a, 2018b; He et al., 2021). Differential accumulation analyses were performed using DEseq2 with default parameters as described in “Materials and methods”. Asterisks indicate *P*-values (corrected for multiple testing) of ≤0.05 (^*^), ≤0.01 (^**^), and ≤0.001 (^***^).

Although we observed that, overall, apoplastic miRNAs were much more abundant outside EVs than inside EVs, this observation did not rule out the possibility that some miRNAs might be specifically loaded into EVs and thus could be enriched inside EVs relative to the general apoplastic miRNA population. To test this hypothesis, we compared the frequencies of individual miRNAs in each sample using a differential gene expression tool (see “Materials and methods”). To avoid false negatives due to low expression, we selected only miRNAs with more than one read per million (RPM) mapped reads in at least one sample. This filter reduced the data set from 427 mature miRNAs to 94. From these, 62 miRNAs displayed differential accumulation in at least one of the comparisons (Figure 4).

Based on the differential accumulation pattern, we placed the miRNAs into one of six clades. Clade I comprised seven miRNAs that were highly accumulated in the trypsin plus RNase A-treated samples compared to control and RNase A alone-treated samples but were not differentially accumulated in RNase A alone treated versus control samples. This is the pattern expected for miRNAs located inside EVs because they should be protected against RNase A degradation regardless of trypsin treatment. Clade II comprised 10 miRNAs that were significantly more abundant in the RNase A alone-treated samples relative to the control samples and in the trypsin plus RNase A-treated samples relative to controls. Three of these also were significantly more abundant in the trypsin plus RNase A samples versus the RNase A-alone samples. This pattern is expected for miRNAs that are located both inside EVs and outside EVs, with the latter being protected against RNase digestion by proteins.

Clades III and VI contained 24 miRNAs that exhibited low accumulation in trypsin plus RNase A-treated samples compared to control and RNase A alone-treated samples, but showed high accumulation in RNase A alone-treated samples compared to the controls. This pattern is expected for miRNAs that are located outside EVs and protected by RNA-binding proteins. The miRNAs found in Clades IV (13 total) and V (8 total) exhibited low abundance in RNase A alone-treated samples versus controls as well as trypsin plus RNase A alone-treated samples versus control samples. These are most likely miRNAs that are located outside EVs and are not protected by proteins. In summary, these data indicate that most plant miRNA species in the apoplast are located outside EVs, with only seven miRNAs apparently enriched inside EVs. Notably, of these seven miRNAs, six correspond to passenger strands of active miRNAs, and thus they might represent unneeded material that is being discarded from the cell.

### Apoplastic tasiRNAs are located mostly outside EVs

tasiRNAs are a subclass of sRNAs that have been proposed to mediate interkingdom RNA interference, possibly by transfer inside of plant EVs (Cai et al., 2018a; He et al., 2021). The analyses presented in Figures 1 and 2, however, indicate that siRNAs are mostly located outside EVs. To determine whether there may be a specific subset of tasiRNAs that are preferentially loaded inside EVs, we performed a differential accumulation analysis of tasiRNAs, as described above for miRNAs. To avoid false positives, we established a minimum cut-off of five RPM in at least one sample, reducing the number from 1581 to 27 tasiRNAs. Of these, all exhibited a differential accumulation that was statistically significant in at least one comparison (Figure 4B). Based on differential abundance in the three samples, we could group these 27 tasiRNAs into five clades.

Clade I (seven tasiRNAs) showed significantly higher relative abundance in RNase A alone-treated samples compared to control samples, suggesting these tasiRNAs are located outside EVs and are protected by proteins. Consistent with this conclusion, these seven tasiRNAs were relatively less abundant in trypsin plus RNase A-treated samples compared to RNase A alone-treated samples. Clade II tasiRNAs (four tasiRNAs) showed a very similar pattern to that of Clade I tasiRNAs, thus are also likely to be located outside EVs and protected by proteins.

Clades III (three tasiRNAs) and IV (eight tasiRNAs) showed a relative abundance pattern opposite to that of Clades I and II—treatment with RNase A alone caused a decrease in relative abundance compared to control samples and treatment with trypsin plus RNase A caused an increase compared to RNase A alone-treated samples. This pattern suggests that tasiRNAs belonging to Clades III and IV are located outside EVs and are not protected by proteins. However, why trypsin plus RNase A treatment led to less efficient removal than RNase A alone is unclear. We speculate that residual trypsin activity in the former may have caused a slight reduction in RNase activity.

Lastly, the five tasiRNAs included in Clade V showed a pattern more similar to RNAs in Clades I and II, suggesting that these are located outside EVs and are mostly protected by proteins. Notably, none of the tasiRNAs showed a pattern that would be consistent with protection inside EVs, as they should show a relative increase in abundance across all three comparisons. It has been previously reported that two tasiRNAs from Arabidopsis, Tas1c-siR483 (here named Tas1c_16_461) and Tas2-siR453 (here named as Tas2_0_566) are transferred into fungal cells via EVs (Cai et al., 2018a). However, in our study, we found that these two TAS-derived siRNAs are present outside EVs, in association with RNA-binding proteins (indicated by red # symbol in Figure 4B).

### AWF contains long RNAs that are protected by RNA-binding proteins

**Figure 5:**
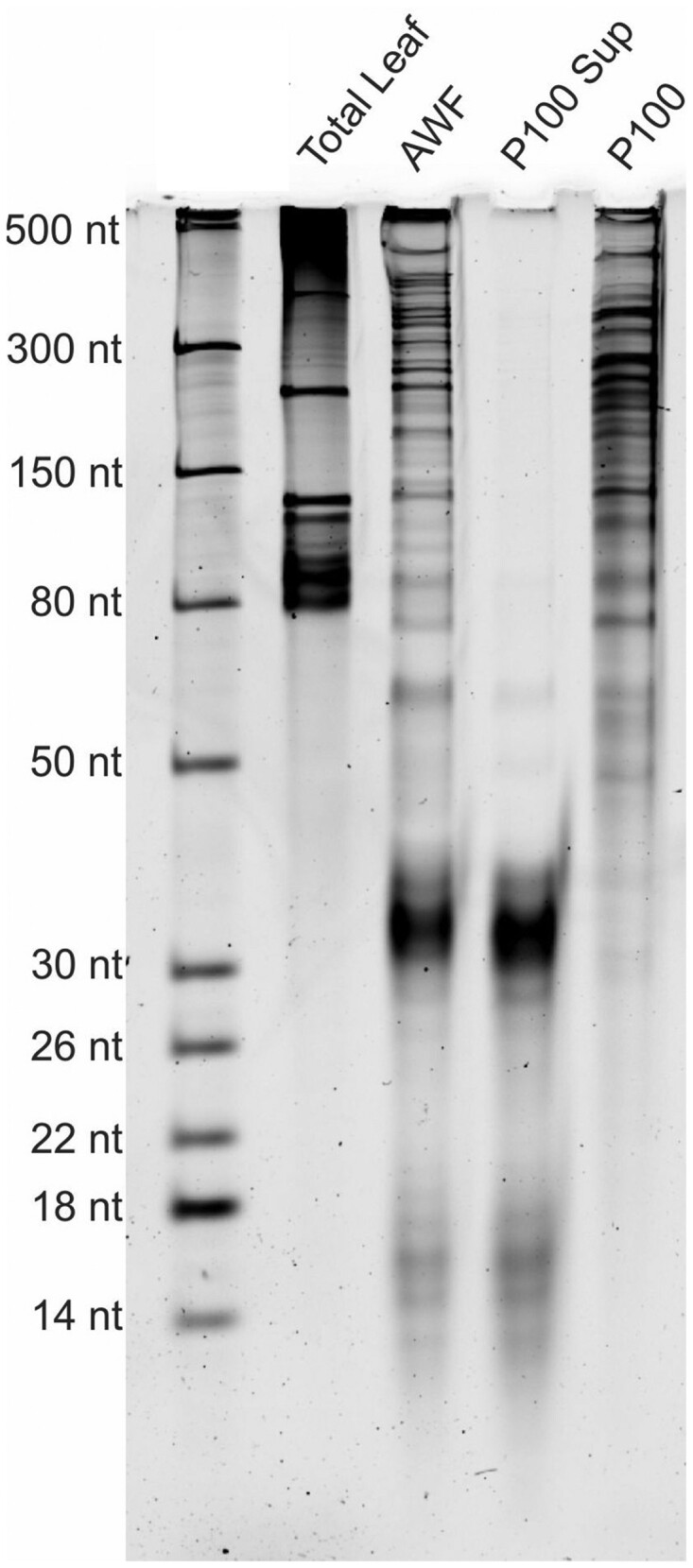
Apoplastic fluid contains long RNAs. Long RNAs are present in AWF and can be pelleted by ultracentrifugation. RNA was isolated from the indicated fractions by TRIzol extraction and then 75 ng of each was separated in a 15% denaturing polyacrylamide gel, followed by staining with SYBR Gold nucleic acid stain. RNA size standards are shown in the left lane. P100 indicates RNA isolated from the pellet obtained after centrifugation at 100,000 *g*. P100 Sup indicates the RNA remaining in the supernatant after the 100,000*g* centrifugation step. Note that the majority of the RNA larger than 50 nt was pelleted at 100,000 *g*, indicating it was associated with particles of some kind. In contrast, the majority of the RNA smaller than 50 nt was not pelleted. This experiment was repeated four times on separate days with independent biological replicates (i.e. different plants), with all replicates producing similar results.

The above analyses revealed that Arabidopsis apoplastic fluid contains sRNA–protein complexes that are located outside EVs, thus defining a new class of exRNA in plants. Recent work in mammalian systems has revealed that mammalian cells secrete lncRNAs independent of EVs (Lasda and Parker, 2016; Preußer et al., 2018). We thus investigated whether plants might also secrete lncRNAs that are extravesicular. For these analyses, we collected AWF from Arabidopsis leaves using the same protocol as used for sRNA isolation (Rutter and Innes, 2017). The resulting AWF was filtered and centrifuged at 100,000 *g*. RNAs were isolated from total AWF, the P100 pellet, and from the supernatant of the P100 pellet and analyzed by denaturing polyacrylamide gel electrophoresis, followed by staining with SYBR Gold to detect nucleic acids. These analyses revealed that AWF contained abundant RNAs ranging in size from 14 nt to at least 500 nt (Figure 5). The pattern of bands observed for the AWF sample was entirely different from the pattern observed for total cellular RNA, indicating that AWF isolation does not cause substantial cell breakage. Notably, the great majority of the RNAs larger than 50 nt were pelleted at 100,000 *g*, while smaller RNAs were not. This observation is consistent with the findings of Baldrich et al (2019), which showed that siRNAs are enriched in the supernatant of P40 pellets compared to EVs or total cellular RNA.

**Figure 6:**
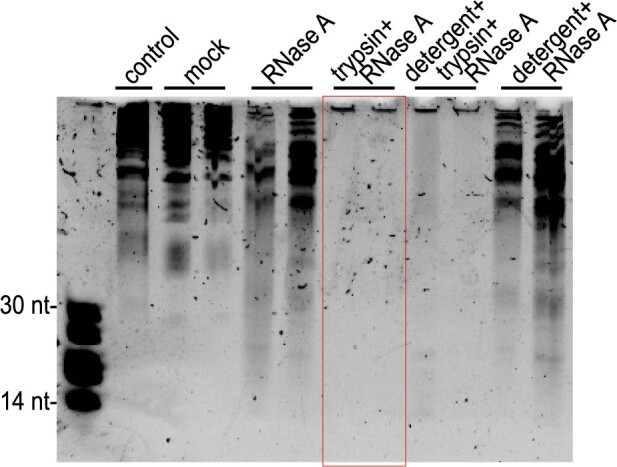
Apoplastic long RNAs are protected against RNase A digestion by proteins. P40 pellets were treated with RNase A, trypsin plus RNase A, Triton X-100 detergent plus trypsin plus RNase A, or detergent plus RNase A. The negative control was input RNA without any treatments and kept on ice. Mock was the same RNA subjected to the same incubations as the treated RNA, but without detergent, RNase A or trypsin. Following these treatments, these RNAs (and size standards, left lane) were separated in a 40% denaturing polyacrylamide gel, followed by staining with SYBR Gold nucleic acid stain. RNA size standards are shown in the left lane. The red box highlights the observation that all RNA was degraded by RNase A treatment following treatment with trypsin, even in the absence of detergent, indicating that the RNA was located outside EVs. This experiment was repeated three times on different days with different source plants and produced similar results.

The observation that RNAs longer than 50 nt pelleted at 100,000 *g* indicated that these RNAs must be associated with some kind of particle, raising the possibility that these RNAs might be carried on or in EVs. To assess whether they were packaged inside EVs, we first treated P40 pellets with trypsin to digest extravesicular proteins and then treated them with RNase A to digest RNAs (Figure 6). Notably, the majority of the P40 RNA was not digested by treatment with RNase A alone, but was completely degraded by treatment with trypsin followed by RNase A. Collectively, these results show that the majority of apoplastic RNAs are located outside EVs but are protected against RNase A digestion by RNA-binding proteins.

### Apoplastic RNA contains circRNAs

**Figure 7:**
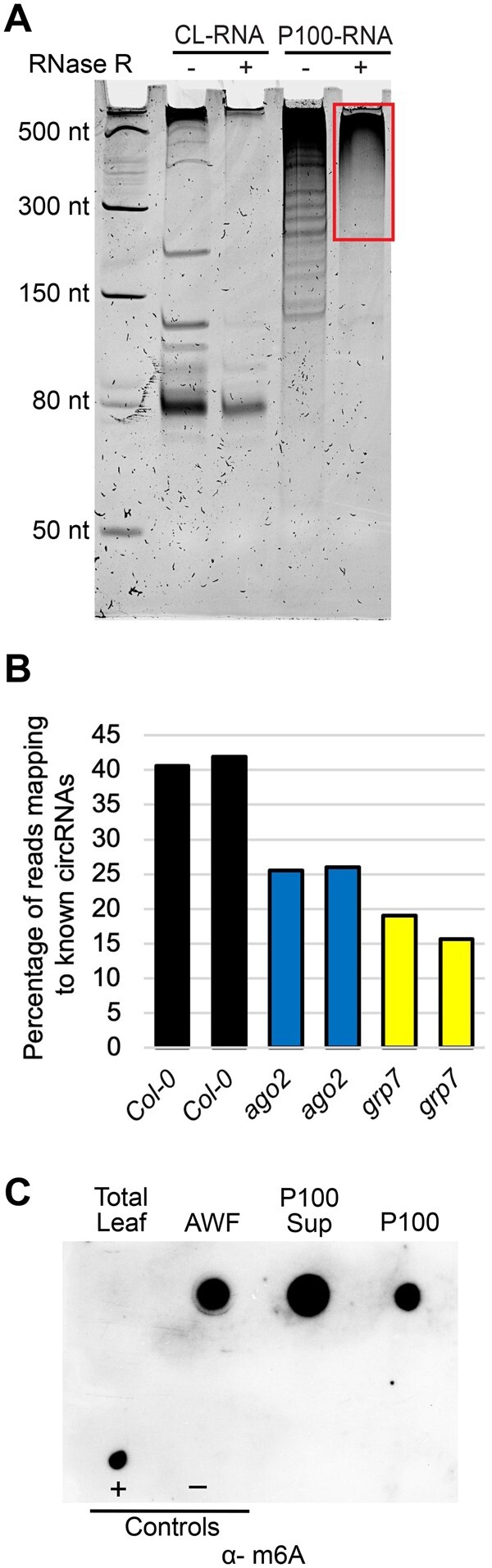
Apoplastic RNAs are enriched in circRNAs and m^6^A-modified RNAs. A, Apoplastic fluid contains circRNAs. Both RNAs from a P100 pellet and RNAs from total CL were purified using our P100 protocol and were then treated with RNase R, which degrades linear RNAs. These RNAs (and size standards, left lane) were then separated in a 15% denaturing polyacrylamide gel, followed by staining with SYBR Gold nucleic acid stain. Red box indicates RNase R-resistant RNA. B, Apoplastic RNA contains diverse circRNAs. P100 RNAs were treated with RNase R to remove linear RNA and then analyzed by RNA-seq using an Illumina NextSeq platform. Graphs indicate the percentage of reads that mapped to known Arabidopsis circRNAs for RNA isolated from wild-type, *ago2* mutant, and *grp7* mutant Arabidopsis plants. Data from two biological replicates (independently isolated P100 pellets) are shown for each genotype. C, Apoplastic RNAs are enriched in m^6^A modification. An aliquot of 200 ng of each of the indicated RNAs used in Figure 5 were dot blotted onto a nitrocellulose membrane and then probed with an anti-m6A antibody. For positive and negative controls, 600 ng of synthetic 21-nt RNAs with identical sequences (except for a single m6A modification on the positive control) were used.

To our knowledge, long exRNAs have not been reported previously in plants. In mammals, however, exRNAs have been extensively characterized due, in part, to their potential use as noninvasive markers for diseases such as cancer (Zhan et al., 2018). Notably, mammalian exRNAs are highly enriched in circRNAs, possibly due to their resistance to digestion by extracellular RNases (Li et al., 2015; Chen and Huang, 2018; Seimiya et al., 2020). To assess whether plant exRNAs also contain circRNAs, we performed RNase R treatment on exRNAs isolated from P100 pellets. This enzyme is a 3′–5′ exoribonuclease that digests most linear RNAs, including RNAs with double-stranded regions such as rRNA, but leaves circRNAs intact (Vincent and Deutscher, 2006). The RNase R-treated RNA was then analyzed by denaturing polyacrylamide gel electrophoresis, revealing that a large amount of RNA larger than 300 nt remained undigested, along with several distinct RNAs shorter than 300 nt (Figure 7). As a control, we homogenized whole Arabidopsis leaf tissue and purified it using our EV isolation protocol. The RNA obtained from this preparation displayed a pattern of RNA bands entirely different from that seen with the P100 RNA, and RNase R treatment eliminated all visible RNA larger than 150 nt. These results indicate that plant exRNA is enriched in circRNAs.

To confirm this conclusion, we generated RNA-seq libraries from P100 RNA that had been treated with RNase R and then mapped the reads from these libraries to a collection of previously identified Arabidopsis circRNAs (Chu et al., 2017), which are defined by the presence of junction fragments derived from back-splicing events (Ye et al., 2019). Consistent with our RNase R analysis, we found that apoplastic RNA contained abundant circRNAs, with over 40% of the reads mapping to known Arabidopsis circRNAs (Figure 7B).

### Apoplastic RNA is enriched in m^6^A modifications

In mammalian systems, circRNA biogenesis often involves posttranscriptional modification with N^6^-methyladenine (m^6^A), which promotes back-splicing, with the resulting circRNAs containing multiple m^6^A-modified sites (Di Timoteo et al., 2020; Zhang et al., 2020a). We thus assessed whether apoplastic RNA might be enriched in m^6^A modification. We isolated RNA from whole leaves, from total AWF, from P100 pellets and from the supernatant of P100 pellets. The concentrations of these RNA preparations were then determined using a NanoDrop spectrophotometer and their concentrations equalized, as shown in Figure 5. RNA samples (200 ng each) were then dot blotted onto a nitrocellulose membrane along with positive and negative control RNAs that consisted of the same 21-nt synthetic RNA, with the positive control containing a single m6A modified nucleotide at position 11. This dot blot was then probed with an anti-m^6^A antibody. This immunoanalysis revealed that exRNA is highly enriched in m^6^A modification relative to total cellular RNA (Figure 7C). Notably, RNAs isolated from both the P100 pellet and the supernatant of the P100 pellet both displayed signals that were stronger than the positive control, suggesting that the density of m6A modification on exRNA is greater than one modification per 21 nt. This observation also indicates that both small exRNAs and long exRNAs are enriched in m^6^A modification.

### Apoplastic RNA is enriched in intergenic RNAs

**Figure 8:**
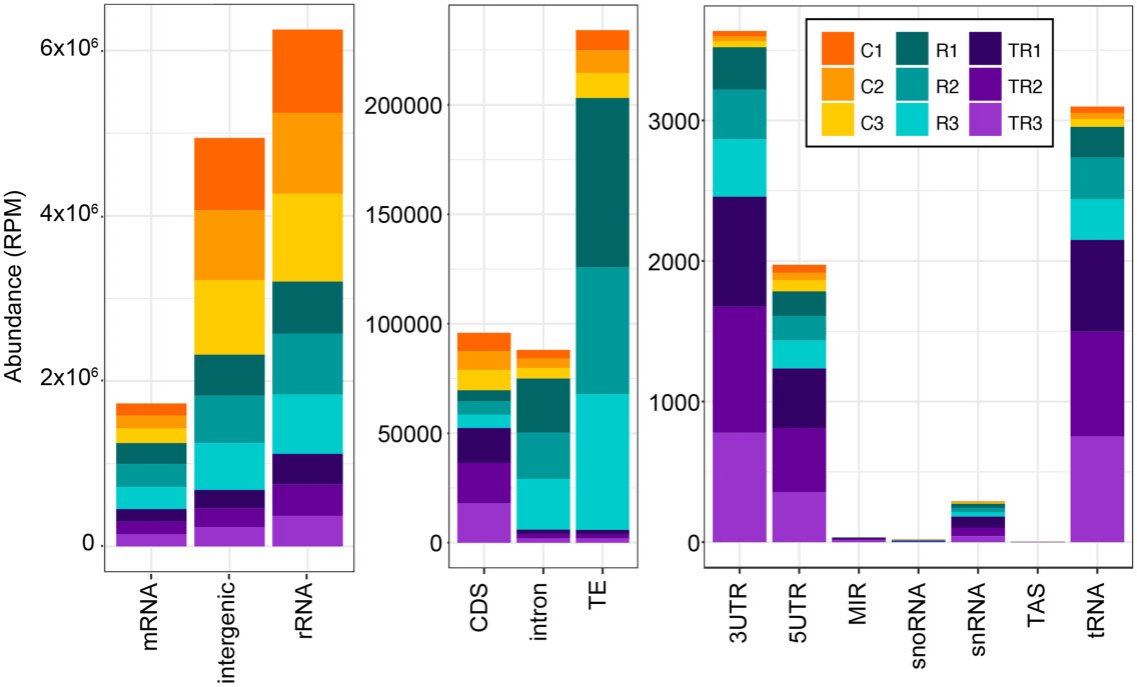
Apoplastic RNA is derived from multiple sources and is enriched in intergenic RNA. RNA-seq reads were mapped to the Arabidopsis genome and categorized as indicated on the *x*-axis and quantified on the *y*-axis as RPM. Note the difference in scales for the three graphs, which were used to better visualize the lower abundance categories. Reads that mapped to protein coding genes (mRNA, left graph) were further categorized as mapping to the 5′-UTR, the 3′-UTR, the protein coding sequence (CDS), or introns. MIR, miRNA encoding gene; snRNA, TAS, trans-acting siRNA-producing loci. The values for three independent biological replicates (P40 pellets isolated from different plants) from each of three treatments are shown. Treatments were: control untreated RNA (C1–C3), RNase A-treated RNA (R1–R3), and trypsin plus RNase A-treated RNA (TR1–TR3).

To determine the sources of apoplastic RNA, we performed Illumina-based RNA-seq analysis on RNA isolated from P40 pellets. We generated two sets of RNA-seq libraries, Method 1 using a poly(A) enrichment protocol, and Method 2 using an rRNA depletion protocol (see Methods). Analysis of the poly(A)-enriched library revealed that it contained very few products with inserts (Supplemental Figure S2), indicating that apoplastic RNA contained very little intact mRNA. This finding also indicates that there was little to no contamination with RNA from broken cells. In contrast, the majority of the products in the second library contained inserts of diverse size, thus was analyzed using Illumina sequencing. Mapping of the resulting reads to the Arabidopsis genome revealed not only that the majority of the reads were derived from ribosomal RNA and intergenic regions but also that they included a large number of reads derived from protein-coding genes (Figure 8). Notably, the latter reads included a large number of reads derived from introns, similar in number to those derived from exons, suggesting that exRNAs are enriched in incompletely spliced or alternatively spliced RNAs. This observation is consistent with the presence of circRNAs, which often include introns.

To assess whether specific RNA species were associated with protein or were encapsulated inside EVs, we also made libraries from P40 pellets that were either treated with RNase A alone, which should eliminate RNA that is not protected by proteins or EVs, or treated with trypsin plus RNase A, which should leave mostly RNA encapsulated in EVs. Analysis of these libraries revealed that trypsin plus RNase A treatment reduced the relative proportion of most classes of exRNA (Figure 8), consistent with our conclusion that the vast majority of exRNAs are located outside EVs but are protected by proteins. Notably, treatment with RNase A alone increased the relative frequency of RNAs that mapped to TEs and introns, which suggests that these RNAs are especially well protected by proteins. In contrast, RNA reads mapping to 5′untranslated regions (5′-UTRs), 3′-UTRs, and tRNAs became relatively more abundant following trypsin plus RNase A treatment (Figure 8), suggesting that these RNAs might be protected inside EVs. We interpret these data with caution, however, as these reads made up a very small fraction of the total reads. It is worth noting, also, that based on paired-end sequence reads, most of the tRNA sequences were derived from tRNA fragments and not full-length tRNAs.

### RNA-binding proteins GRP7 and AGO2 are secreted into the apoplast Independent of EVs

The above analyses revealed that AWF contains abundant RNA species (including both sRNAs and long RNAs) that are protected from RNase degradation by proteins. This raised the question of what RNA-binding proteins are present in the apoplast. In our previous proteomic analyses of density-gradient purified EVs, we had identified the RNA-binding protein GLYCINE-RICH PROTEIN 7 (GRP7) as copurifying with EVs (Rutter and Innes, 2017). GRP7 has two RNA-binding domains and binds to multiple species of RNA, including sRNAs, precursors of miRNAs and pre-mRNAs (Streitner et al., 2012; Nicaise et al., 2013; Köster et al., 2014). Arabidopsis GRP7 has been shown to participate in plant responses to pathogen infection (Fu et al., 2007; Lee et al., 2012; Nicaise et al., 2013). In addition, it is targeted by the bacterial type III-secreted effector HopU1, which blocks the interaction between GRP7 and GRP7-associated mRNAs, resulting in a reduction in translation of defense-related proteins (Nicaise et al., 2013). It has also been shown that Arabidopsis GRP7 regulates alternative splicing of pre-mRNAs and directly binds to pre-mRNAs, modulating alternative splicing (Streitner et al., 2012). All of these observations made GRP7 a prime candidate for further analysis with regard to its role in exRNA production and/or accumulation.

**Figure 9:**
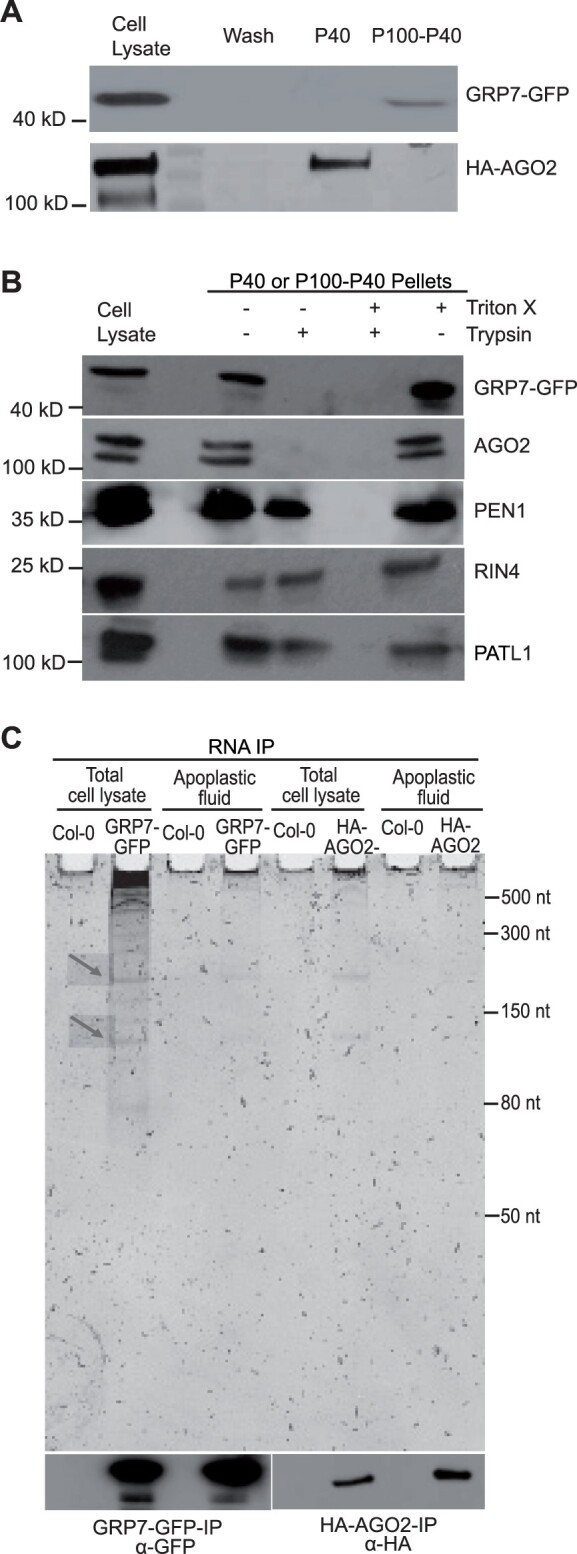
GRP7 and AGO2 are secreted to the apoplast independent of EVs and bind to lncRNAs. A, GRP7 and AGO2 are present in the apoplast. Apoplastic fluid was isolated from HA-tagged AGO2 and GFP-tagged GRP7 transgenic Arabidopsis plants and was then pelleted at 40,000 *g* (P40) followed by another round of centrifugation at 100,000 *g* (P100-P40). Apoplastic HA-AGO2 mostly pelleted at 40,000 *g*, whereas GRP7-GFP mostly pelleted at 100,000 *g* (P100-P40). Wash; 40 μL of apoplastic wash prior to ultracentrifugation. B, GRP7 and AGO2 are located outside EVs. GRP7- and AGO2-containing pellets were treated with trypsin with or without detergent. GRP7 and AGO2 were eliminated even in the absence of detergent, whereas known EV cargo proteins PEN1, RIN4 and PATL1 were not eliminated. C, GRP7 and AGO2 bind to lncRNAs. RNAs isolated from GRP7-GFP-RNAIP and HA-AGO2-RNAIP were separated in a 15% denaturing polyacrylamide gel, followed by staining with SYBR Gold. Nontransgenic wild-type Arabidopsis was used as a negative control.

To confirm that GRP7 is secreted into the apoplast, we performed immunoblots on protein extracts isolated from the P40 and P100-P40 fractions of an Arabidopsis line expressing GRP7-GFP expressed under its native promoter (Figure 9). These analyses revealed that GRP7 was mostly found in the P100-P40 fraction, and therefore likely was not located inside EVs, which mostly pellet in the P40 fraction. To confirm that GRP7 was located outside EVs, we performed a protease protection assay. GRP7 was degraded in the absence of detergent, indicating that it was located outside EVs (Figure 9B).

In parallel to our analyses on GRP7, we also assessed whether the sRNA-binding protein ARGONAUTE2 (AGO2) was present in AWF. AGO2 was chosen from among the ten Arabidopsis AGO proteins because of (1) a known role in plant-pathogen interactions (Harvey et al., 2011) and (2) the availability of a high-quality commercial antibody from Agrisera. Our analyses revealed that, like GRP7, AGO2 is also present in the apoplast, with the majority of it located outside EVs (Figure 9, A and B).

### GRP7 and AGO2 associate with lncRNAs in the apoplast

To investigate the RNAs associated with GRP7 and AGO2 in the apoplast, we performed RNA immunoprecipitation on whole cell lysate (CL) and P100 fractions. RNAs were separated by size using polyacrylamide gels, followed by staining with SYBR Gold to detect nucleic acids. These analyses indicated that, in both whole CL and in the P100 fraction, Arabidopsis GRP7-GFP expressed under the native GRP7 promoter binds to RNAs of various sizes ranging from 50 nt to more than 500 nt (Figure 9C). Similarly, immunoprecipitation of Arabidopsis HA-AGO2 expressed under its native promoter revealed that Arabidopsis AGO2 binds to lncRNAs in both CL and extracellular spaces of plant cells (Figure 9C). In mammalian systems, it has been reported that AGO proteins can bind to lncRNAs through AGO/miRNA complexes (Tarallo et al., 2017). Recently, mammalian AGO proteins have been shown to bind to circRNAs and may function in loading circRNAs into the extracellular matrix (Hansen et al., 2011; Chen et al., 2019; Xu et al., 2020a). The interaction between circRNAs and AGO might be mediated by miRNAs or through interaction with another RNA-binding protein that binds to circRNAs (Hansen et al., 2011; Chen et al., 2019; Zang et al., 2020).

### Mutation of *AGO2* or *GRP7* alters apoplastic circRNA content

To investigate whether AGO2 and/or GRP7 exert a specific effect on circRNA secretion or stability in the apoplast, we performed RNA-seq analyses on exRNAs from *grp7* and *ago2* mutants following RNase R treatment. These analyses revealed a marked reduction in total circRNAs identified in each mutant (Figure 7B), suggesting that these proteins contribute to circRNA secretion or stabilization.

## Discussion

Prior to the work presented above, it was unclear whether siRNAs and miRNAs found in the apoplast of plant leaves are primarily packaged inside EVs or are exported via an alternative pathway. In our previous work, we had shown that removal of EVs from AWF does not deplete the fluid of most siRNAs, suggesting that most siRNAs are located outside EVs (Baldrich et al., 2019). However, Cai et al. (2018a) reported that siRNAs pellet with plant EVs and are resistant to degradation by micrococcal nuclease. Based on these observations, it was concluded that these siRNAs were packaged inside EVs. To address these seemingly contradictory results, we treated EV pellets with protease plus RNase A, which should eliminate sRNAs located outside EVs but not those located inside EVs. The majority of sRNAs in the size classes of 21, 22, and 24 nt were eliminated (Figure 1A), indicating that most siRNAs and miRNAs are not located inside EVs but instead are located outside EVs and are protected from nucleases by RNA-binding proteins. This finding is consistent with recent work in mammalian systems showing that many sRNAs that copurify with EVs can be digested with protease plus RNase treatment (Shurtleff et al., 2017; Jeppesen et al., 2019) and are thus likely located outside EVs. This finding also suggests that EVs may not play a direct role in translocating sRNAs into other organisms such as fungal pathogens. Instead, it appears that sRNA–protein complexes located outside EVs could be the primary mediators of interkingdom RNA silencing.

Although our data indicate that the majority of sRNAs are located outside EVs, it is important to note that many sRNAs pellet with EVs during differential ultracentrifugation. This could be because the sRNAs are bound to protein complexes of a size similar to that of EVs and/or they could be associated with the surface of EVs. EVs have a relatively high surface area in comparison to their volume, which can promote interactions between EVs and other extracellular molecules (Janas et al., 2015; Buzás et al., 2018). A tight association between sRNAs and EV surface proteins could potentially protect sRNAs from degradation by nucleases.

In addition to sRNAs, our analyses of apoplastic RNAs revealed that plants secrete lncRNAs into the extracellular space. Although it has been reported that some extracellular lncRNAs are located inside mammalian EVs (Takahashi et al., 2014; Chen et al., 2016; Zheng et al., 2018; Dai et al., 2020), our data indicate that extracellular lncRNAs produced by plants are located outside EVs and are associated with RNA-binding proteins. As with sRNAs, we found it was necessary to treat apoplastic pellets with protease prior to RNase A to determine whether lncRNAs were inside or our outside EVs, as treatment with RNase A alone had very little effect (Figure 6A).

In mammalian systems, lncRNAs have been shown to regulate multiple biological processes, including gene transcription (Luo et al., 2016), translation (Hu et al., 2018; Jia et al., 2019), and epigenetic modifications (Neumann et al., 2018), as well as cell-to-cell communication (Wei and Wang, 2015; Cai et al., 2018b; Zhu et al., 2021). lncRNAs have also been shown to contribute to antiviral innate immune responses in mammalian systems (Ouyang et al., 2016; Liu et al., 2020). Similarly, lncRNAs in plants have also been shown to modulate gene expression, epigenetic regulation and response to stresses (Di et al., 2014; Wang et al., 2018; Hamid et al., 2020; Moison et al., 2021). However, the presence of lncRNAs in the extracellular space of plant cells and their roles in cell-to-cell communication or immune responses have not been investigated. Whether plant extracellular lncRNAs can be taken up by pathogen cells is unknown, but the ability of fungi to take up long single-stranded and double-stranded RNAs in a Petri dish suggests that this is likely (Qiao et al., 2021). If so, it will be interesting to assess whether these RNAs can impact gene expression in fungi and other plant-associated organisms.

A subclass of lncRNAs of particular interest is circRNAs, as these RNAs not only were previously shown to be induced by pathogen infection in plants but also appear to contribute to immunity (Fan et al., 2020). Our sequencing data revealed that Arabidopsis exRNA contains thousands of circRNAs. At the same time, no intact full-length mRNAs were identified, indicating that circRNAs are preferentially secreted or are more stable in the apoplast than linear mRNAs. This finding is similar to that reported for cultured human cells, in which circRNAs were found to copurify with EVs and to be highly enriched relative to their matching linear RNAs found in CLs (Lasda and Parker, 2016).

Extracellular circRNAs in mammals have been suggested to contribute to cell-to-cell communication (Lasda and Parker, 2016). One likely function of mammalian extracellular circRNAs is to sequester miRNAs (Hansen et al., 2013). Whether plant circRNAs play a similar role in the apoplast is not known, but it is tempting to speculate that they could function as target mimics for sRNAs secreted by pathogens. Pathogens have been reported to deliver sRNAs into plant cells to suppress immunity and enhance susceptibility (Weiberg et al., 2013; Wang et al., 2017; Dunker et al., 2020). Therefore, it could be quite useful to have a collection of circRNAs in the apoplast to sequester sRNAs secreted by pathogens before they can reach their targets inside the host cell.

The discovery that plants accumulate lncRNAs in their extracellular spaces raised the fundamental question of how this RNA is secreted. We found that the RNA-binding proteins AGO2 and GRP7 also accumulate in the apoplast and are bound to lncRNAs. Elimination of these proteins altered the RNA content of the apoplast, indicating a possible function of AGO2 and GRP7 in the secretion of RNA into the apoplast or the stabilization of RNAs once there. Notably, GRP7 belongs to the same family of RNA-binding proteins as human HNRNPA2B1, which has been shown to mediate sorting of specific miRNAs into EVs (Villarroya-Beltri et al., 2013), and to bind to m^6^A-modified RNA (Alarcón et al., 2015). This suggests that GRP7 is fulfilling similar roles in plants. Consistent with this hypothesis, we found that plant exRNAs are highly enriched in m^6^A modifications. Whether m^6^A modification plays a role in the secretion of exRNAs into the apoplast and/or contributes to their stability requires further investigation.

## Materials and methods

### Plant materials and growth conditions

The *A.* *thaliana grp7* mutant (SALK_039556.21.25.x) was obtained from the Arabidopsis Biological Resource Center at Ohio State University. The Arabidopsis *ago2-1* mutant was obtained from James Carrington at the Donald Danforth Plant Science Center. The Arabidopsis HA-AGO2 transgenic line was also obtained from Dr Carrington. It expresses HA-AGO2 under the native *AGO2* promoter in an *ago2-1* mutant background (Montgomery et al., 2008). The GRP7-GFP transgenic line was obtained from Dr Dorothee Staiger at Bielefeld University. This line expresses GRP7-GFP under control of the native *GRP7* promoter and contains the *GRP7* 5′-UTR, intron and 3′-UTR in a *grp7–1* mutant background (Köster et al., 2014). Seeds were germinated on 0.5 × Murashige and Skoog medium containing 1% agar (Murashige and Skoog, 1962). To induce synchronous germination, Petri dishes containing the seeds were stored at 4°C for 2 days and then moved to short-day conditions where they were illuminated using GE HI-LUMEN XL Starcoat 32-watt fluorescent bulbs (a 50:50 mixture of 3,500 and 5,000 K spectrum bulbs) with 9 h days, 22°C, 150 µEm^−2^s^−1^. After 10 days, the seedlings were transferred to Pro-Mix PGX Plug and Germination Mix with Biofungicide supplemented with Osmocote slow-release fertilizer (14-14-14), both of which were obtained from Hummert International (St Louis, MO, USA). Seedlings were grown under a clear plastic dome for the first week following transfer.

### Isolation of EVs and other apoplastic particles

For a single biological replicate, AWF was isolated from 24 6-week-old Arabidopsis plants as described in Rutter and Innes (2017). Briefly, Arabidopsis rosettes were vacuum-infiltrated with vesicle isolation buffer (VIB), pH 6.0, containing 20-mM 2-(*N*-morpholino) ethanesulfonic acid, 2-mM CaCl_2_, and 0.01-M NaCl as described previously (Rutter et al., 2017). After vacuum infiltration, the excess buffer was removed from leaf surfaces by blotting rosettes with Kimwipes. To recover apoplastic fluid from infiltrated leaves, rosettes were placed inside needleless, 30-mL syringes (two rosettes per syringe). Syringes were placed inside 50-mL tubes and centrifuged for 20 min at 700 *g* with slow acceleration (4°C, JA-14 rotor, Avanti J-20 XP Centrifuge; Beckman Coulter, Indianapolis, IN, USA). The AWF was then filtered through a 0.22 µm membrane (Acrodisc syringe filter, Pall Corporation, New York, USA) and centrifuged at 10,000 *g* for 30 min to remove any remaining large particles. The supernatant was transferred into new centrifuge tubes and centrifuged at 40,000 *g* (P40) or 100,000 *g* (P100) for 1 h (4°C, TLA100.3, Optima TLX Ultracentrifuge; Beckman Coulter) to pellet EVs and other particles as noted in figure legends. The pellet was washed and pelleted again at 40,000 *g* or 100,000 *g* at 4°C using a TLA100.3 rotor, Optima TLX Ultracentrifuge (Beckman Coulter). The pellets were resuspended in 100 µL of cold and filtered VIB (0.22 µm) and either used immediately or stored at −80°C until further use.

### RNA purification

Total leaf RNA was isolated from 100 mg of fresh or frozen leaf tissue using TRIzol Reagent (Thermo Fisher Scientific, Waltham, MA, USA). Briefly, to isolate RNA, leaf tissue was frozen in liquid nitrogen and ground into powder using a mortar and pestle. One milliliter of TRIzolReagent (Thermo Fischer Scientific, Waltham, MA, USA) was added to the ground leaf tissue and mixed vigorously by vortexing. The leaf and TRIzol mixture was then shaken at room temperature for 10 min, followed by the addition of 200 µL of chloroform. This mixture was then vortexed for 30 s and then centrifuged at 12,000 *g* for 15 min. The aqueous phase was removed and mixed with one volume of cold isopropanol to precipitate the RNA. RNA pellets were washed using cold 80% ethanol. To isolate RNA from P40 and P100 pellets, 1 mL of TRIzol was added to 100 µL of resuspended pellet, followed by the same procedure as used for leaf RNA isolation. To isolate RNA either from the supernatant of P100 pellets, or from total AWF (i.e. prior to centrifugation at 100,000 *g*), the RNA was precipitated by mixing with 0.1 volume of 3-M sodium acetate (pH 5.2) and 1.0 volume of cold isopropanol, incubated at −20°C for 1 h and then centrifuged at 12,000 *g* at 4°C for 30 min. The resulting pellet was then resuspended in 1 mL of TRIzol followed by the same procedure as used for leaf RNA isolation. RNA pellets were resuspended in 10–12 µL of ultrapure DNase/RNase-free water (Invitrogen, Waltham, MA, USA) and stored at −80°C. The RNA quality and quantity was assessed using either a ThermoFisher NanoDrop One spectrophotometer or an Agilent 2200 Tape Station.

### Trypsin and RNase A treatments

To assess whether RNAs were located inside or outside EVs, we performed RNase protection assays as follows. P40 pellets were treated with 1-µg/mL trypsin (Promega, Madison, WI, USA) in the presence or absence of 1% (v/v) Triton X-100 (EMD-Millipore, Burlington, MA, USA) in 15-mM Tris–HCl (80-µL final volume). Samples were incubated at 37°C for 1 h followed by addition of 1.5 µg/mL trypsin inhibitor (Worthington Biochemical. Corp, Lakewood, NJ USA) to inactivate trypsin. For the RNase-treated samples, RNase A (Qiagen, Hilden, Germany; diluted in 15-mM NaCl, 10-mM Tris–HCl pH 7.5) was added to the mixture to a final concentration of 5 µg/mL (100-µL final volume) and the sample was incubated at room temperature for 30 min. Immediately after RNase A treatment, RNA was isolated using 1 mL of TRIzol as described above. To inhibit RNase A activity, a mixture of 10 µg/mL RNase Inhibitor, Murine (APExBIO) and 40 units/mL of RNase Out (Invitrogen) was added to the RNAs, which were stored at −80°C until library preparation.

### Immunoblots

For immunoblots, 30 µL of P40 suspensions were combined with 10 µL of 4 × SDS loading buffer (250-mM Tris–HCl, pH 6.8, 8% (w/v) sodium dodecyl sulfate, 40% (v/v) glycerol, 20% 2-mercaptoethanol and 0.004% (w/v) bromophenol blue) and were then heated at 95°C for 5 min. Leaf lysate samples were used as positive controls. The lysate was prepared by freezing 100 mg of leaf tissue in liquid nitrogen and grinding with a mortar and pestle. Ground leaf tissue was extracted in 800 µL of protein extraction buffer (150-mM NaCl, 50-mM Tris–HCl, pH 7.5, 0.1% (v/v) Nonidet P-40, 1% (w/v) 2,2'-dipyridyldisulfide, and 1% plant protease inhibitor cocktail [Sigma-Aldrich, St Louis, MO, USA ]) and centrifuged at 10,000 *g* for 10 min at 4°C to pellet cell debris. An aliquot of 30 µL of leaf lysate was combined with 10 µL of 4 × SDS loading buffer, and the mixture was heated at 95°C for 5  min. Then, 40 µL of P40 and 4 µL of leaf lysate were loaded onto gradient gels (4%–20% Precise Protein Gels, Thermo Scientific) and were separated at 150 V for 1 h in Laemmli electrophoresis running buffer (24.8 mM Tris base, 0.1% (w/v) sodium dodecyl sulfate, 192 mM glycine, pH 8.3). After the proteins were transferred to a nitrocellulose membrane (GE Water & Process Technologies), membranes were washed with 1× Tris-buffered saline (50-mM Tris–Cl and 150-mM NaCl, pH 7.5) containing 0.1% Tween-20 (TBST) and blocked with 10% Difco Skim Milk (BD) in TBST for 1.5 h at room temperature. Membranes were incubated overnight at 4°C with the following primary antibodies at the indicated dilutions: rabbit polyclonal anti-PEN1 (Zhang et al., 2007; 1:1,000), rabbit polyclonal anti-GRP7 (Streitner et al., 2008; 1:1,000), rabbit polyclonal anti-PATL1 (Peterman et al., 2004; 1:5,000), rabbit polyclonal anti-RIN4 (Arabidopsis Biological Resource Center catalog number AB00040; 1:2,000), rabbit polyclonal anti-TET8 (PhytoAB catalog number PHY1490A; 1:1,000), mouse monoclonal [9F9.F9] anti-GFP (Abcam, Cambridge, UK, catalog number ab1218; 1:2,000), rabbit polyclonal anti-AGO2 (Agrisera catalog number AS13 2682; 1:1,000); peroxidase conjugated rat monoclonal [3F10] anti-HA (Roche, catalog number 12013819001; 1:3,000). Filters were then washed with TBST, and if needed, incubated with one of the following secondary antibodies as appropriate: peroxidase-conjugated goat anti-rabbit (Abcam, catalog number ab97051; 1:10,000) or peroxidase-conjugated goat anti-mouse (Abcam, catalog number ab6789; 1:5,000) for 1.5 h at room temperature. After a final wash in TBST, proteins were visualized using ProtoGlow ECL Substrate (National Diagnostics) and a ChemiDoc Imaging System (Bio-Rad, Hercules, CA, USA) and/or X-ray film.

### RNA-immunoprecipitation (RNA-IP)

To isolate RNAs associated with GRP7-GFP and AGO2-HA in whole leaves and in apoplastic fluid of transgenic Arabidopsis plants, we performed RNA-immunoprecipitation **(**RNA-IP). For leaves, we used 1 *g* of fresh or frozen leaf tissue, which was frozen under liquid nitrogen and ground with a mortar and pestle. Leaf powder was mixed with 5 mL of cold IP buffer (0.05 M Tris–HCl, pH 7.4, 0.1 M KCl, 2.5 mM MgCl_2_, 0.1% NP-40, 1% Triton X-100 and 50 U/mL RNase Out), incubated on ice for 10 min, and then transferred to a 15-mL polypropylene screw-cap centrifuge tube. The tube was then centrifuged for 10 min at 12,000 *g* and the supernatant was filtered through a 0.45  µm membrane (Acrodiscsyringe filter, Pall Corporation). The filtered supernatant was then incubated for one h at 4°C with 50 µL of anti-GFP agarose beads (Chromotek) to precipitate GRP7-GFP and with 50 µL of anti-HA agarose beads (Thermo Fisher) to precipitate AGO2-HA. Beads were then pelleted by centrifugation at 1,000 *g* for 2 min at 4°C and washed at least six times with 5 mL of cold IP buffer for 5 min at 4°C for each washing step. Finally, beads were washed two times with 1.5 mL of cold IP buffer followed by a final wash with 1 mL of ultrapure RNase-free/DNase-free water before pelleting by centrifugation at 538 *g* for 1 min. To immunoprecipitate GRP7-GFP and AGO2-HA from apoplastic fluid, P100 pellets were resuspended in 2 mL of cold IP buffer and proteins immunoprecipitated as described for whole leaf extracts.

To isolate RNA, beads were incubated with proteinase K (Invitrogen) at a final concentration of 1.5 µg/µL in 100 µL of PK buffer (0.1 M Tris–HCl, pH 7.4, 0.01 M EDTA, pH 8.0, 300 mM NaCl and 2% SDS) for 1 h at 55°C with intermittent shaking (every 3 min for 15 s). Beads were pelleted by centrifugation at 538 *g* for 1 min, and RNA isolation was performed using the TRIzol reagent as described above.

### Polyacrylamide gel preparation and electrophoresis

RNA samples were analyzed using denaturing polyacrylamide gel electrophoresis. Gels containing 15% polyacrylamide and 7 M urea were prepared using IBI InstaPAGE 40% acrylamide solution (37.5:1). RNA samples were denatured at 65°C in denaturing buffer (0.25 M EDTA (pH 8.0), 8-M urea, 0.2-mg/mL bromophenol blue, 0.02-mg/mL xylene cyanol) and were then separated on 0.5 × Tris-Boric Acid EDTA (0.5 × TBE; 0.065 mM Tris (pH 7.6), 21 mM boric acid, 1.25-mM EDTA)-15% polyacrylamide urea gels. For size standards, we used New England Biolabs Low Range ssRNA Ladder (catalog number N0364S) and Takara 14-30 ssRNA Ladder Marker (catalog number 3416). SYBR Gold Nucleic Acid Gel Stain (ThermoFisher) was used to stain gels for 30 min before UV transillumination. Gel images were acquired using a Bio-Rad ChemiDoc-MP imaging system.

### RNA dot blots using anti-m^6^A antibodies

RNA was isolated from leaf or apoplastic P40 and P100 fractions using TRIzol as described above, and the RNA concentrations were measured using a ThermoFisher NanoDrop One spectrophotometer. For all samples, equal amounts of RNA were prepared in equal volumes (6 µL) using UltraPure DNase/RNase-free distilled water (Invitrogen. RNA samples were denatured at 95°C for 3 min and placed on ice immediately to prevent the formation of secondary structures. RNA samples were applied directly to a piece of Hybond-N+ membrane (Amersham Pharmacia Biotech) using a micropipettor. To prevent the spread of RNA on the membrane, 2 µL of RNA solution was applied at a time, allowing the membrane to dry for three min before applying the next 2 µL drop to the same spot, until a total of 6 µL of RNA sample was applied. To crosslink the spotted RNAs to the membrane, an UVC-508 Ultraviolet Cross-linker (Ultra-Lum) was used to irradiate the membrane twice at 120,000 microjoules/cm^2^ for 30 s. The membrane was then washed in clean RNase-free 1 ×  PBS buffer (1 × PBS; 2.7 mM KCl, 8 mM Na_2_HPO_4_, 2-mM KH_2_PO_4_, and 137-mM NaCl, pH 7.4) and blocked in 5% nonfat milk in 1 × PBS containing 0.02% Tween-20 for 1 h at room temperature. The membrane was then incubated overnight with anti-m^6^A antibody (Abcam catalog number ab151230) at a 1:250 dilution in 5% nonfat milk in 1 × PBS containing 0.02% Tween-20. The membrane was washed in 1 × PBS containing 0.02% Tween-20 three times and incubated with horseradish peroxidase-labeled goat anti-rabbit antibody (Abcam catalog number ab205718) at a 1:5,000 dilution for 1 h. After a final wash in 1 ×  PBS contain 0.02% Tween-20, m^6^A modified RNAs were visualized using the Immune-Star Reagent (Bio-Rad) and imaged using X-ray film.

### Preparation of circRNA samples

To determine the presence of circRNAs, RNA was isolated from the entire P100 pellet (resuspended in 100-µL VIB) obtained from 24 Arabidopsis plants using a PicoPure RNA isolation kit (Thermo Fisher). The RNA (1–3 µg) was then treated with 5 units of RNase R (Lucigen. RNR07250) for 1 h at 37°C. To visualize circRNAs, the RNA samples were separated on denaturing TBE-15% polyacrylamide urea gels and stained with SYBR Gold. To prepare RNA libraries for sequencing, it was necessary to remove RNase R from the RNA samples, and this was accomplished by repurifying the RNase R-treated RNA samples using a PicoPure RNA isolation column.

### Preparation of sRNA-seq and RNA-seq libraries

sRNA libraries were constructed using the RealSeq-AC kit (no. 500-00048; RealSeq Biosciences, Santa Cruz, CA, USA) following the manufacturer’s recommendations. To capture all types of sRNAs, we used 1 µg of RNA as starting material. Except for RNase-R treated samples, all RNA-seq libraries were generated using the NEBNext Ultra II Directional RNA Library Prep Kit for Illumina (catalog number E7765; New England Biolabs) using 500 ng of total RNA as the starting material. The rRNA was removed using the RiboMinus Plant Kit for RNA-Seq (catalog number A1083808, Thermo Fisher Scientific) and poly(A) RNA purification was attempted using the NEBNext Poly(A) magnetic isolation module (catalog number E7490, New England Biolabs). For sequencing of RNase R-treated samples, RNA-seq libraries were prepared using an Illumina TruSeq Stranded mRNA Library Prep kit (catalog number 20020594; Illumina) following the manufacture’s protocol except for skipping the poly(A) enrichment step. All libraries were sequenced on an Illumina NextSeq 550 instrument with paired-end 75-bp reads, except for the RNase R-treated samples, which were sequenced using paired-end 300-bp reads. Sequencing was performed at the Center for Genomics and Bioinformatics at Indiana University, Bloomington, IN, USA.

### Data analysis

Sequences in the sRNA sequencing libraries were trimmed of adaptors using the software Cutadapt version 1.16 (Martin, 2011) using a minimum insert size of 10 nt and a maximum of 34 nt. Sequence quality was assessed using FastQC (http://www.bioinformatics.babraham.ac.uk/projects/fastqc/). Clean reads were aligned to the Arabidopsis genome (TAIR version 10), and all subsequent analyses were performed using the software Bowtie2 (Langmead and Salzberg, 2012). For miRNA analyses, the latest version of miRBase (version 22; (Kozomara and Griffiths-Jones, 2014) was used. Sequences in the RNA-seq libraries were also trimmed of adaptors using Cutadapt version 1.16 (Martin, 2011) and sequence quality was assessed using FastQC. Clean reads were aligned to the Arabidopsis genome (TAIR version 10), using HISAT2 version 2.2.1 (Kim et al., 2019). To identify circRNAs, mapping was performed using the Arabidopsis data on PlantcircBase version 5.0 (http://ibi.zju.edu.cn/plantcircbase/) (Chu et al., 2017). We only considered reads mapping concordantly and exclusively to the junction part of the circRNA. Differential accumulation analyses were performed using DESeq2 with default parameters, using reads that were not normalized as input (Love et al., 2014). In DESeq2, p-values were calculated using the Wald test and corrected for multiple testing using the Benjamini and Hochberg procedure (Benjamini and Hochberg, 1995). Graphical representations were generated using the software ggplot2 (Wickham, 2009) in the R statistical environment.

### Accession numbers

The data discussed in this publication have been deposited in NCBI's Gene Expression Omnibus (Edgar et al., 2002) and are accessible through GEO Series accession numbers GSE183867 (https://www.ncbi.nlm.nih.gov/geo/query/acc.cgi?acc=GSE183867) and GSE185133 (https://www.ncbi.nlm.nih.gov/geo/query/acc.cgi?acc=GSE185133). The accession numbers for Arabidopsis proteins discussed in this work are AT1G48410 (AGO1), AT1G31280 (AGO2), AT2G21660 (GRP7), AT1G72150 (PATL1), AT3G11820 (PEN1), AT1G59870 (PEN3), and AT3G25070 (RIN4).

## Supplemental data 

The following materials are available in the online version of this article.

**Supplemental Figure S1.** Apoplastic miRNAs and tasiRNAs are mostly located outside EVs and are protected by proteins.

**Supplemental Figure S2.** P40 RNA appears to lack polyadenylated RNA.

## Supplementary Material

koac043_supplementary_data

## Notes

H.Z.K., P.B., B.D.R., R.W.I., and B.C.M. designed the research; H.Z.K., P.B., B.D.R., K.Z., and L.B. performed the research; P.B. analyzed the sRNA-seq and RNA-seq data; H.Z.K., P.B., and R.W.I. wrote the article; all authors read and commented on the article.

The author responsible for distribution of materials integral to the findings presented in this article in accordance with the policy described in the Instructions for Authors (https://academic.oup.com/plcell) is: Roger W. Innes (rinnes@indiana.edu).
