# Supplementary material for: Arabidopsis apoplastic fluid contains sRNA- and circular RNA–protein complexes that are located outside extracellular vesicles
Source: Plant Cell. 2022 Feb 16;34(5):1863–81. doi: 10.1093/plcell/koac043 (PMC9048913; doi:10.1093/plcell/koac043)
Supplement: koac043_supplementary_data [file koac043_supplementary_data.pdf]

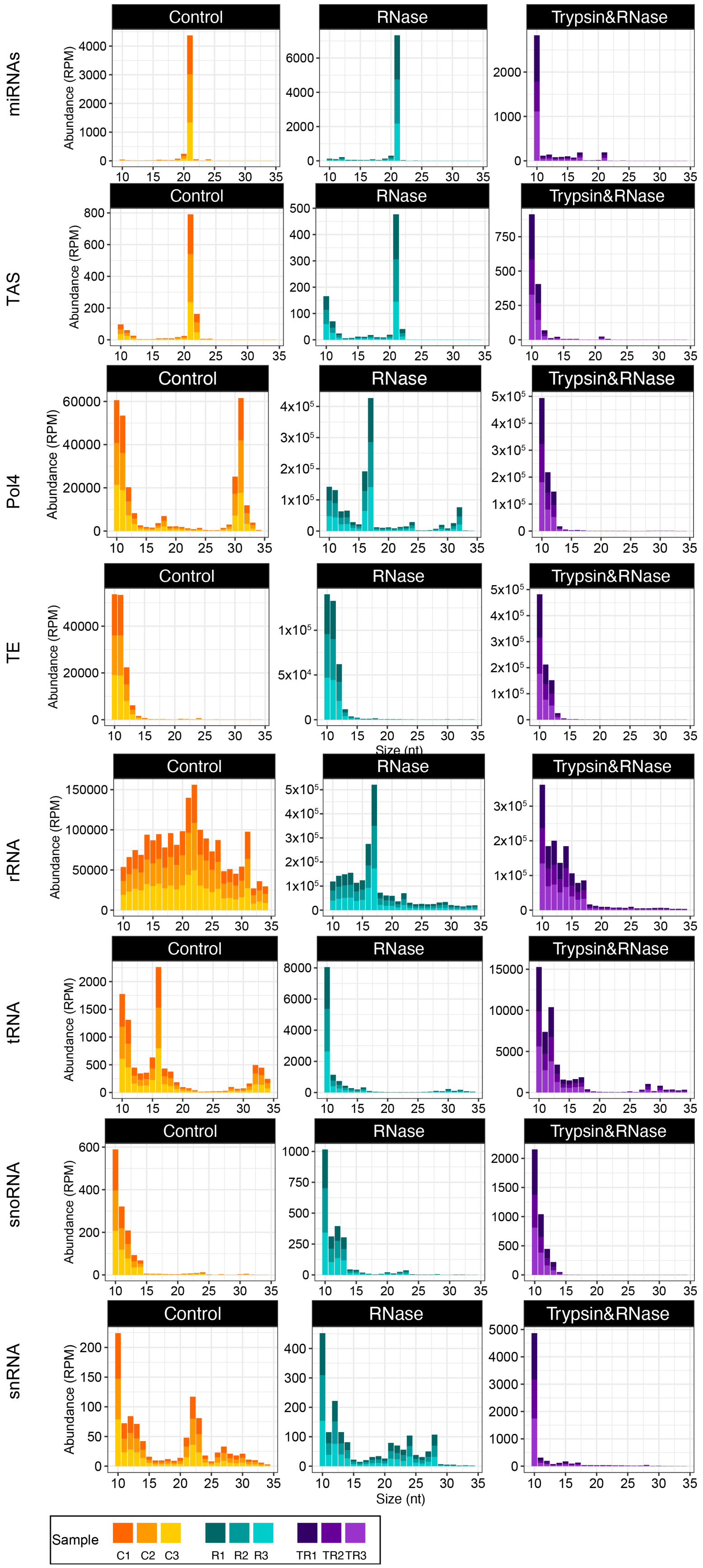

Supplemental Figure S1.
Apoplastic miRNAs and trans-acting siRNAs are mostly located outside EVs and are protected by proteins. (Supports Figure 2)

Graphs indicate the size distributions of P40 sRNAs mapping to the indicated sources. The abundance of each size class was calculated for each P40 treatment: Control, RNase A only, and trypsin plus RNase A. The x axis indicates the sRNA size, and the y axis indicates its abundance in reads per million mapped reads (RPM). Data from three independent biological replicates are stacked together in a single bar plot and color coded as shown in the legend.

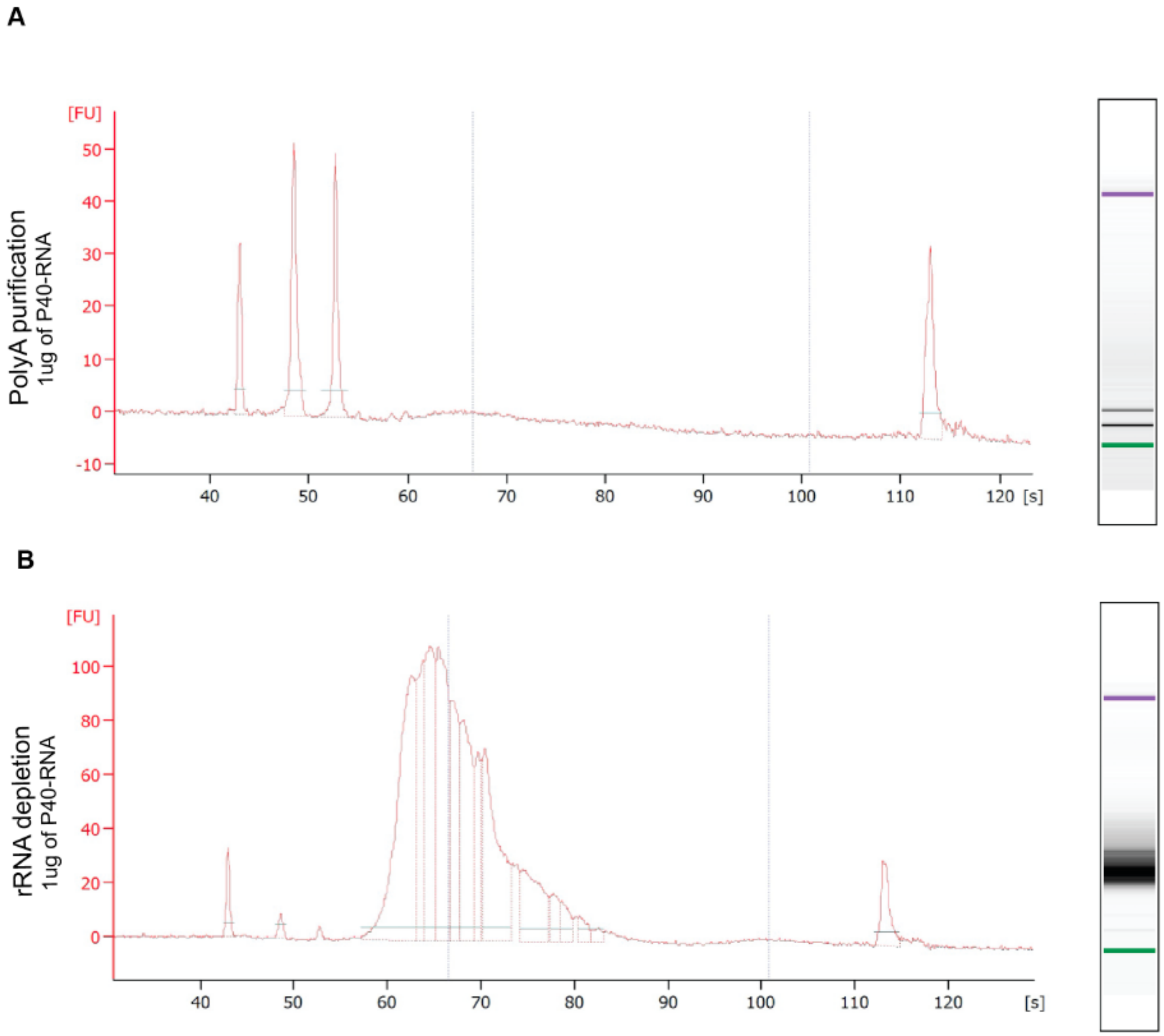

Supplemental Figure S2. P40 RNA appears to lack polyadenylated RNA.
(Supports Figure 8)

RNA-seq libraries were prepared from P40 RNA using two different methods.
(A) Method 1 used a poly(A) enrichment step to specifically copy polyadenylated mRNAs. Analysis of the sizes of the inserts in the resulting library using an Agilent Tape Station revealed that most products lacked an insert, indicating a lack of full-length mRNAs in the P40 fraction.

(B) The second method used a ribosomal RNA depletion step, but no poly(A) enrichment step. This library produced products with the expected size range of inserts (note the broad peak between 60 and 80 seconds).
